# Immobilization of cadmium and lead in contaminated paddy field using inorganic and organic additives

**DOI:** 10.1038/s41598-018-35881-8

**Published:** 2018-12-13

**Authors:** Yasir Hamid, Lin Tang, Xiaozi Wang, Bilal Hussain, Muhammad Yaseen, Muhammad Zahir Aziz, Xiaoe Yang

**Affiliations:** 1 Ministry of Education (MOE) Key Laboratory of Environmental Remediation and Ecosystem Health, College of Environmental and Resources Science, Zhejiang University, Hangzhou, 310058 People’s Republic of China; 2 Institute of Soil and Environmental Sciences, University of Agriculture, 38080 Faisalabad, Pakistan

## Abstract

Heavy metal contamination of agricultural soils has posed a risk to environment and human health. The present study was conducted to assess the effectiveness of soil amendments for reducing cadmium (Cd) and lead (Pb) uptake by rice (*Oryza sativa* L) in a contaminated field. The soil amendments used include lime, DaSan Yuan (DASY), DiKang No.1 (DEK1), biochar, Fe-biochar, Yirang, phosphorus fertilizer, (Green Stabilizing Agent) GSA-1, GSA-2, GSA-3, and GSA-4, applied at 1% rate in a field experiment. The results exposed that GSA-4 treatment showed best effects on reducing Cd and Pb phytoavailability in soil and uptake by early rice. Linear increase in pH (i.e. 5.69 to 6.75) was recorded in GSA-4 amended soil from sowing to the 3rd month of growth season. GSA-4 decreased DTPA extractable contents of cadmium (Cd) from 0.324 to 0.136 mg kg^−1^ soil and lead (Pb) from 53.21 to 24.68 mg kg^−1^ soil at 90 days of amendment. Treatment with GSA-4 improved rice growth (56%) and grains yield (42%). The enhancement effects on grain yield may be result from the positive effects of GSA-4 application on increasing photosynthesis (116%) and transpiration rate (152%) as compared to the control. Significant reduction in Cd and Pb uptake in shoot (42% and 44%) and in grains (77 and 88%), was observed, respectively in GSA-4 treatment as compared with the control. Moreover, negative correlation was recorded between DTPA extractable Cd/Pb and soil pH that directly depended on applied amendments. In short, use of combined amendment (GSA-4) was more effective for immobilizing heavy metals in contaminated paddy field, and secures rice safe production, as compared other tested amendment products.

## Introduction

Heavy metals (HMs) enter soil environment via different anthropogenic activities e.g., smelting, mining, disposing hazardous materials and fertilization^1–3^. Metals contamination to soil has been associated with industrial emissions and disposal of wastes in agricultural lands. Heavy metals accumulation through food chain has adverse effect on human health^4,5^. Among trace metals Cd and Pb are most phytotoxic that inhibits plant growth and enter food chain through plant uptake from contaminated soil^6^. Cadmium and Pb are categorized as most hazardous metals by United States Environmental Protection Agency (US-EPA)^7^. Mostly, metals are non-biodegradable, so their availability severely affects plants growth, soil quality and humans health. There is a dire need of cost effective and environmental friendly techniques to reduce metals accumulation in food chain^8^.

Metals availability reduction is the key for remediation of HMs contaminated soils. Different practices e.g. physical, biological and chemical and phytoremediation have been adopted to remediate metals polluted sites^9^ but most techniques are not efficient in terms of time and cost. *In-situ* remediation of HMs with different kinds of soil amendments (organic, inorganic and clay minerals) is gaining much attention in the last decade. Amendments reduce metal risk and uptake by plants and offsite contamination via leaching. However, organic amendments especially manures and biochar enhanced heavy metal immobilization by increasing soil pH, CEC and providing adsorption sites to bind metals^10,11^. Organic materials make soluble or insoluble complexes with heavy metals that decreases metal uptake by plants, which ultimately reduces risk to food chain. Organic materials like green manure, animal wastes and composts can effectively remediate contaminated sites by transforming exchangeable/soluble fraction to organic bound or residual fraction, which are less available to plants^12^. Organic materials are also good source of essential nutrients; improve soil fertility and microbial interactions in soil^13^. *In-situ* immobilization by clay minerals such as sepiolite and zeolite has been reported for reduced heavy metals availability^14,15^.Clay minerals are abundant in nature with highly negative charged layer to adsorb cations. Minerals act as sorption agents due to hydroxyl group presence and are effective in reducing heavy metal availability through adsorption or complexation^16,17^. This adsorption process facilitates to reduce metal leaching in soil profile^18^. Heavy metals bioavailability is largely pH dependent, higher in acidic conditions than alkaline, so application of liming material can neutralize acidic soil and enhance metal stabilization in soil^19^. Alkaline materials act as pH regulating mediators and increase metals precipitation with help of hydroxyl and carboxyl groups or by providing more adsorption sites by causing deprotonation on soil surface^20^. Different lime based materials e.g. calcium carbonate (CaCO_3_), burnt lime (CaO) and dolomite (CaMg(CO_3_)_2_) have been extensively used to stabilize HMs in soil^20^.

Several studies have been reported about individual outcome of organic, inorganic and clay minerals on heavy metals sorption in soil but their combined impact on immobilization of heavy metals in field conditions is not well accredited. So, this field experiment was designed with objectives: (1) comparing the effectiveness of different commercial soil immobilizing products on reducing Cd and Pb uptake by early rice; (2) understanding the metal response to pH change with different time intervals; (3) identify best effective amendment for early rice growth and safe production.

## Results and Discussions

### Effects on available Cd and Pb in soil

**Figure 1:**
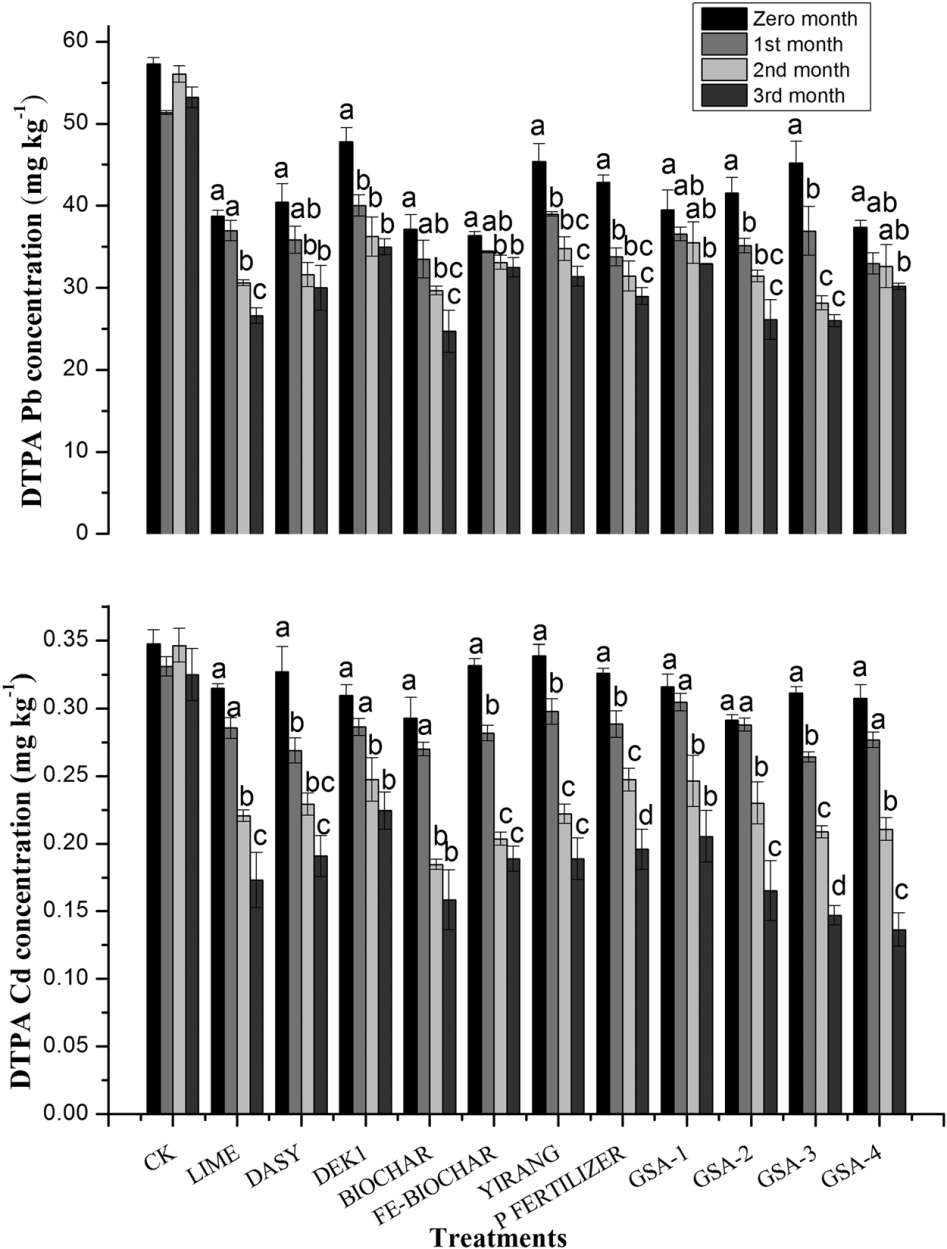
Effect of different amendments on available heavy metals concentration in soil under field conditions. All Amendments were applied at 1% concentration level. All values are averaged of 3 replicates (n = 3).

Cd and Pb availability was monitored from amendment application to the 3rd month of crop growth (Fig. 1). After three month of soil amendments, DTPA extractable Cd in soil was reduced from 0.324 (control) to 0.136 mg kg^−1^ soil (GSA-4) and up to 0.147 mg kg^−1^ soil in treatment received GSA-3. In general, organic amendments can reduce Cd availability by adsorption or complexation^21,22^. It is obvious from the results that application of organic material does not affect total Cd content but decrease Cd availability by converting exchangeable fraction to organic matter bound fraction^23^. Biochar reduced metal availability by increasing surface sorption on biochar. Possible mechanisms of reduced metal phytoavailability by biochar application include precipitation of metal-organo compounds^24^. Lime application decreased metal mobility through attributes of precipitation and adsorption processes by changing soil pH. An increase in pH of paddy soil was observed with sepiolite application and this elevation in soil pH results in precipitation of metals as metal hydroxides and/or carbonates^20^. Similar reaction might occur to biochar treatment, which reduced Cd availability from 0.324 to 0.158 mg kg^−1^ soil. Overall reduction in Cd availability among the treatments decreased in the order of GSA-4 > GSA-3 > Biochar > GSA-2 > Lime > FE-BIOCAHR > Yirang > DASY > P fertilizer > GSA-1 > DEK1 > CK (0.136; 0.147; 0.158; 0.165; 0.173; 0.188; 0.188; 0.190; 0.195; 0.205; 0.224; 0.324 mg kg^−1^ soil, respectively).

A decrease in Pb availability was observed in all the treatments. After three months of amendment, biochar decreased DTPA extractable Pb from 53.2 (control) to 24.68 mg kg^−1^ soil in GSA-4 treatment. Treatments GSA-3 and GSA-2 resulted in reduction of DTPA extractable Pb by 25.98 and 26.12 mg kg^−1^ soil, respectively. Biochar has been widely used to remediate HMs contaminated soils and proposed mechanisms include ion exchange, adsorption, precipitation and co-precipitation^25–27^. Alkaline treatment (Liming) significantly decreased DTPA extractable Pb (26.58 mg kg^−1^), as compared to control, which can be attributed to pH increase^28^ and consequently enhanced metal precipitation^29^. The overall reduction in DTPA extractable Pb decreased in the following order for the different treatments: biochar > GSA-3 > GSA-2 > Lime > P fertilizer > DASY > GSA-4 > Yirang > Fe-biochar > GSA-1 > DEK1 > CK (24.68; 25.98; 26.12; 26.58; 28.95; 29.98; 30.16; 31.38; 32.48; 32.88; 34.96; 53.21 mg kg^−1^ soil).

### Effect of amendments on soil pH

**Figure 2:**
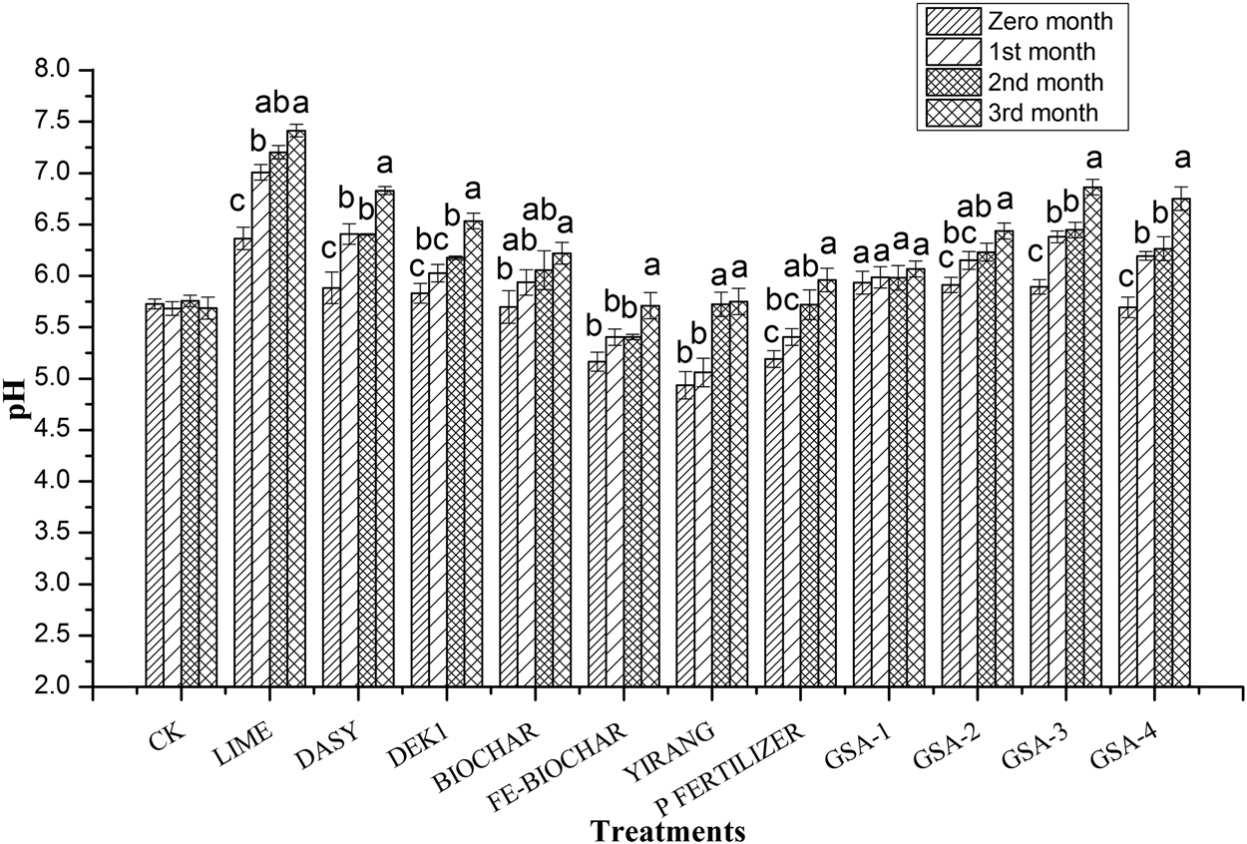
Effect of different amendments on soil pH with advance of rice growth stages. All Amendments were applied at 1% concentration level. All values are average of 3 replicates (n = 3). Bars sharing the same letters in one treatment are statistically non-significant at 5% significance level.

A rapid increase in soil pH was observed in all the treatments in 15 days after amendments application except for Fe-biochar, Yirang and P fertilizer (Fig. 2). Soil liming caused consistent increase in soil pH from start to 3rd month of time interval of crop. There was significant difference in pH with lime as compared to control and other treatments. In three months, liming increased soil pH from 5.69 to 7.41. This increase in pH with liming resulted in improved nutrients availability and crop growth. Alkaline amendments are known for raising soil pH and surface negative charge, thus reducing heavy metal activity by precipitation and ion adsorption^30^. There was a small increase in soil pH (5.71, 5.75, 5.96), with Fe-biochar, yirang and P fertilizer at 3rd month of treatments but not significantly different from control. Application of GSA-3 and GSA-4 also raised soil pH to a noticeable level (6.86, 6.75). Addition of organic materials like manure and wood powder can change soil properties (pH, EC) that affect HMs availability due to elevated soil pH^31^.

**Figure 3:**
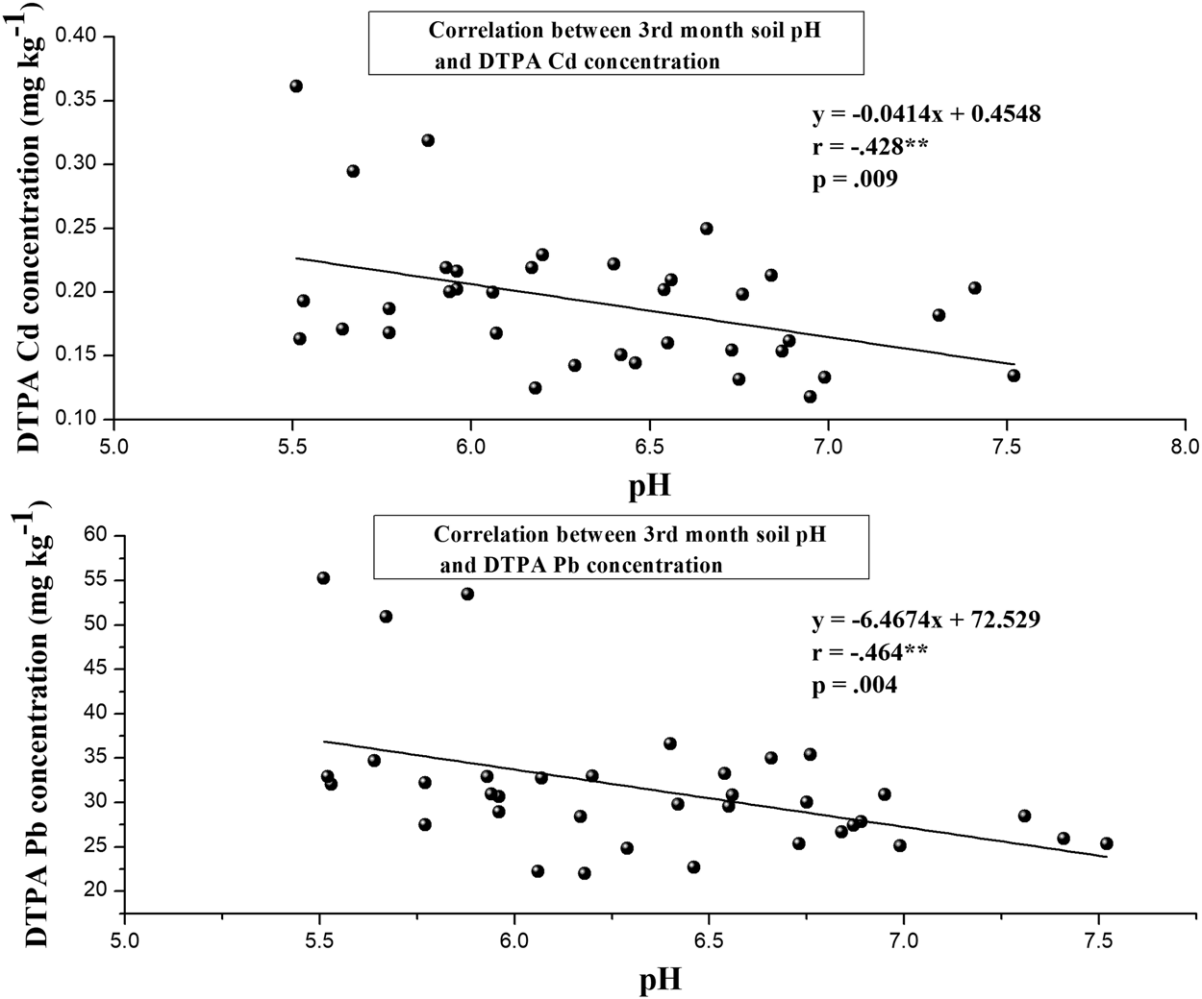
Correlation coefficients between soil pH and available Cd and Pb concentrations after different soil amendments application for 3rd month.

There was significant negative correlations between DTPA extractable Cd or Pb and soil pH at the three-month treatments (Fig. 3), with a correlation coefficients of −0.428** and −0.464**, respectively. This correlation indicated that pH is an important soil property that affects heavy metals availability in contaminated soils^32,33^.

### Effects on metal concentration and uptake

**Figure 4:**
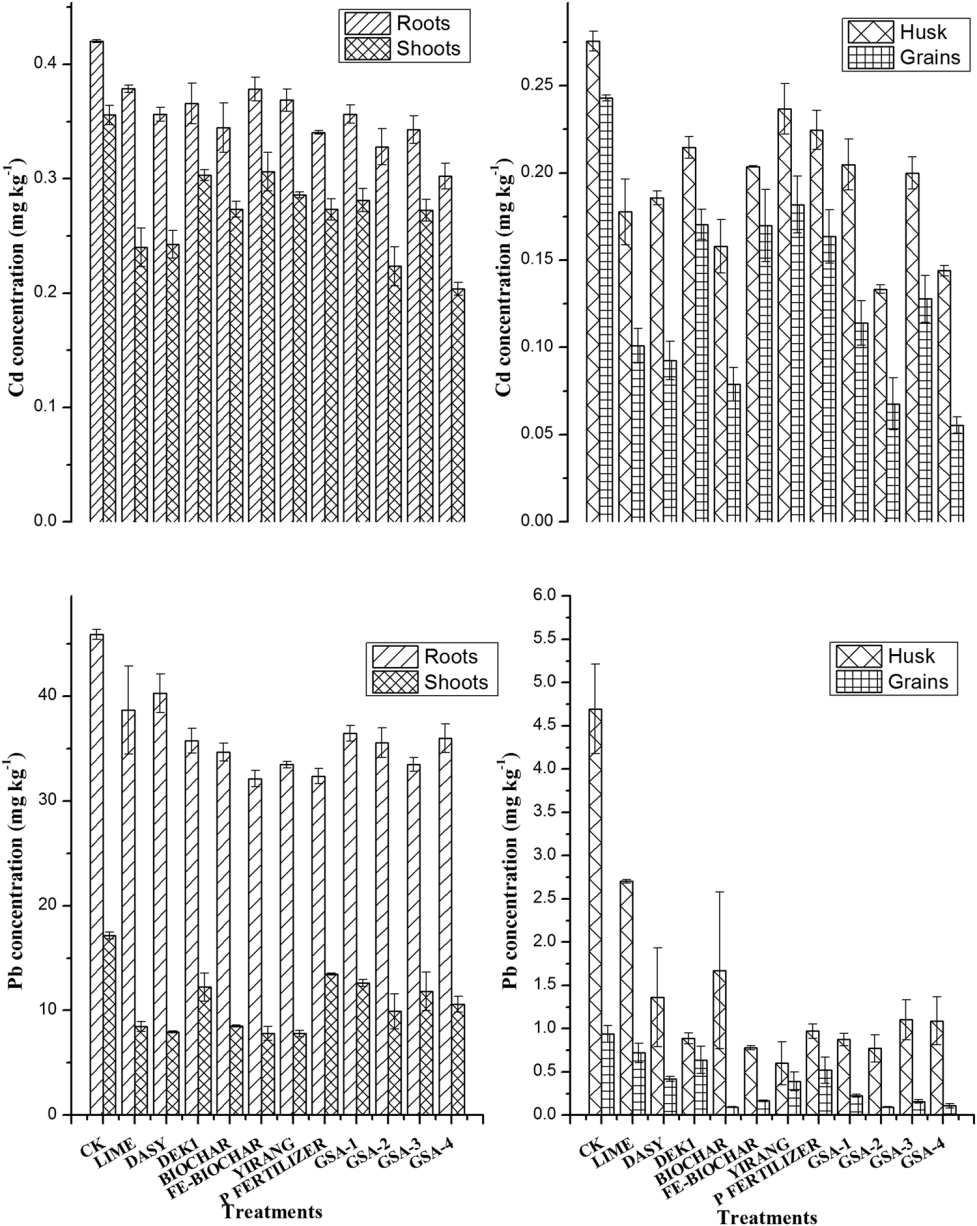
Effect of different amendment on cadmium and lead concentration in rice grain and other plant parts. Plants were harvested after maturity. All data were means of three replications; bars indicate standard errors of the mean value. The differences among the treatments were analyzed by the LSD method.

Cadmium and Pb concentration in plant parts such as roots, shoots, grains and husk generally decreased with application of amendments as compared to control (CK) (Fig. 4). Despite small changes in pH with Fe-biochar, Yirang and P fertilizer application, these treatments showed a reduction in plant concentrations of heavy metals. Concentration of Cd and Pb in rice roots was higher than other parts and no significant difference was observed among all the treatments. Previous studies indicated that Cd contamination effect on rice growth depends on total Cd contents, soil physico-chemical properties, rice species and water management^34^. Cadmium concentration in roots, shoots, husk and grains were 0.302, 0.203, 0.1439, and 0.055 mg kg^−1^, respectively in the composite treatment (GSA-4) where manure, lime and sepiolite were applied in combination, which is a significant decrease, as compared to control (Fig. 4). Application of biochar alone and in combination with lime, sepiolite and zeolite (GSA-2) reduced bioaccumulation of Cd in rice grains 67% and 72%, respectively. The availability and mobility of HMs in soil is dependent on organic source, clay minerals, soil pH and Fe/Al oxides. Organic amendment provides a novel environmental friendly option in reducing trace metal accumulation in edible parts of plants. Adsorption, complexation and/or precipitation are the possible mechanisms involved in immobilization of metals by organic amendment^35^. Organic matter has reactive groups (carboxyl and hydroxyl) that can react with soluble cadmium to form stable complexes^8^. Overall, shoot Cd concentration was significantly decreased by the composite additives (GSA-4, GSA-2), as compared to CK (0.203; 0.223 mg kg^−1^, respectively). These composite treatments reduced shoot Cd contents by 42% and 37%. These results revealed the effectiveness of combined organic and inorganic sources in reducing Cd bioaccumulation in rice, which can be attributed to the decreased Cd availability in soil. Application of biochar alone and combined with inorganic and mineral additives (GSA-2) reduced Pb uptake by rice. Lead concentration in rice grains was significantly decreased in GSA-2, biochar and GSA-4 (90, 89 and 88%) treated plots respectively as compared to control. Addition of biochar in contaminated soil increased soil pH and reduced phytoavailability of metals. The potential role of biochar in reduced Cd uptake is more effective than other fixing agents^36^. The reduction in Cd and Pb contents in rice may be owing to improved immobilization by biochar treatment and changes in soil HMs mobility. Combined application of alkaline material and sepiolite is well reported for elevated soil pH and heavy metals reduction in different crops^37^. This increase in pH level may result a decrease in trace metals mobility and enhanced immobilization^38^. Alkaline treatment (lime) did not show significant difference in grains Pb contents (0.719 mg kg^−1^) with control. Overall reduction in Pb contents of rice grains was as GSA-2 > biochar > GSA-4 > GSA-3 > Fe-biochar > GSA-1 > Yirang > DASY > P fertlizer > DEK1 > Lime > CK (90; 89; 88; 83; 82; 76; 58; 55; 44; 32; 23%). Shoot Pb contents were lessened in Yirang, Fe-biochar and DASY treated plots (54; 54 and 53%). Biochar may act as liming material with consistent increase in soil pH due to its alkaline nature^39,40^. This soil pH elevation by biochar amendment endorses metals adsorption and complexation on biochar particles due to high affinity of biochar for cation exchange capacity.

### Effects of soil amendments on rice grains and biomass yield

Rice grains yield and biomass was determined at harvesting from 1 square meter area of each treatment (Table 1). The application of GSA-4 significantly increased fresh weight of biomass (2.03 kg m^−2^), as compared to control (1.69 kg m^−2^). Combined amendment of biochar, lime, sepiolite and zeolite (GSA-2) also resulted in a significant increase in biomass fresh weight (2.01 kg m^−2^) than other treatments. Biological yield was also increased up to 20300 and 20133 kg ha^−1^ by the application of amendments GSA-4 and GSA-2, respectively over control (16966 kg ha^−1^). As compared to control (5611 kg ha^−1^), per hectare grain yield was more in GSA-3 (8037 kg ha^−1^) in which a combination of organic and inorganic additives was applied. Composite treatment (GSA-4, GSA-2) also significantly improved grains yield of early rice (7913 and 7787 kg ha^−1^). Animal manure and biochar are effective soil amendments that are commonly used for enhancing crop production by improving soil structure and nutrient availability^41–43^. Biochar is also regarded as soil conditioner as its application increases soil fertility and plant growth by supplying retaining nutrients and changing soil physic-chemical properties^44^. Addition of manures increases soil organic matter contents, CEC and pH buffering capacity, and soil physical properties^45^. Liming is helpful in increasing pH of acidic soil, thus reducing the activity of hazardous elements in soil. This decreases the activity of contaminants in soil that reduced phytotoxicity which ultimately leading to a significant increase in yield^46^. It has been reported that mixture of lime and phosphate amendments increased rice biomass in Cd contaminated field^47^. Liming was also testified to sustain crop yield in Cd contaminated soil^48^. The role of natural clay minerals (sepiolite, zeolite) has already been conferred for remediation of HMs contaminated sites due to their easy availability and low cost^49^. Application of sepiolite at 10 g kg^−1^ soil increased crop production by 65%, as compared to control^50^, likely due to stabilization of heavy metals in the contaminated soil^51^.

**Table 1:** Effect of soil amendments on growth parameters of rice.

Treatments	Biological yield (kg ha^−1^)	Grains yield (kg ha^−1^)
CK	16966d	5611b
LIME	18866a-d	7209ab
DASY	17200cd	7118ab
DEK1	17066cd	7378ab
BIOCHAR	18800a-d	7197ab
Fe-BIOCHAR	18700a-d	7131ab
YIRANG	17733b-d	7041ab
PF	19333a-d	7314ab
GSA-1	19466a-c	7491ab
GSA-2	20133ab	7787a
GSA-3	19433a-c	8037a
GSA-4	20300a	7913a

Note: All values are averaged of 3 replicates (n = 3). Different letters indicates significantly different values at 5% significance level, PF = Phosphorus fertilizer. All treatments including control receive recommended dose of N, P and K except treatment 8 in which phosphorus was not applied.

### Effect of amendments on leaf photosynthesis of rice plants

**Figure 5:**
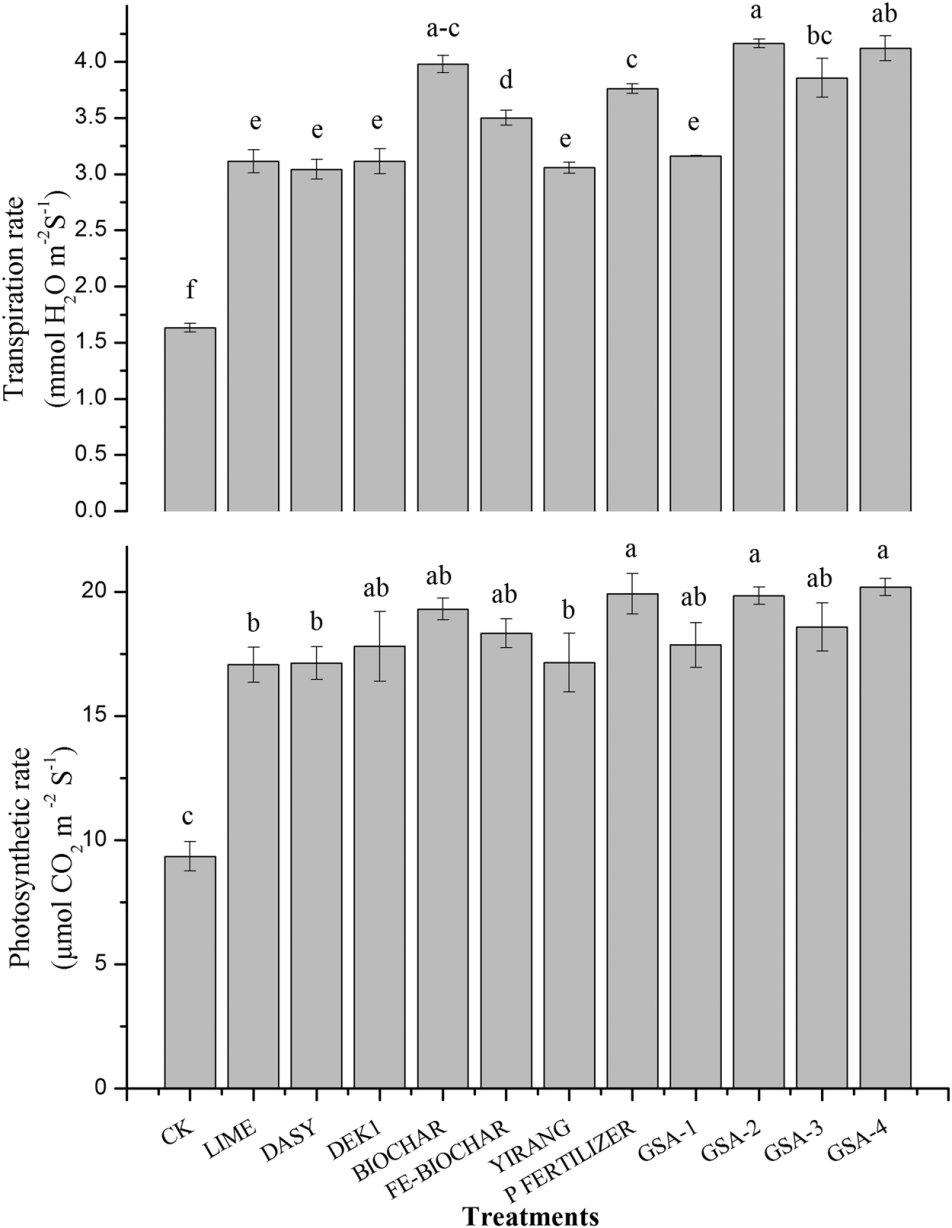
Effect of application of different amendments on leaf photosynthesis (μmol CO_2_ m^−2^ S^−1^) and transpiration rate (mmol H_2_O m^−2^ S^−1^) in rice. Bars with different letters represent significant difference among treatments. All values are average of 3 repeats (n = 3).

Composite treatments (GSA-4 and GSA-2) and application of single P fertilizer showed significant increase in photosynthetic rate (Fig. 5). Up to 116 and 112% increase in photosynthetic rate was recorded in treatments GSA-4 and GSA-2, respectively, as compared to control (CK). Similarly composite treatments GSA-2, GSA-4 and single biochar significantly increased transpiration rate over control (Fig. 5). Maximum (154%) increase in transpiration rate was observed in GSA-2 treatment where biochar was applied in composite with inorganic sources, followed by GSA-4 and biochar (152 and 143%, respectively over control). Several stress conditions were responsible for reduction in plant physiological processes in HMs contaminated soil. Heavy metals toxicity negatively affected gas exchange attributes like photosynthetic and transpiration rate in different plants and caused adverse effect on plant photosynthetic apparatus and mineral nutrient balance^52^. Addition of amendments can improve plant growth by increasing nutrient supply and reducing metal contact with plants^53^. This increase in physiological properties may also due to reduced uptake and accumulation of metals in plants. An increase in photosynthetic rate with biochar application was previously reported^54^. A significant increase in photosynthetic rate and transpiration rate was noted with biochar application under stress conditions in maize and wheat^53^.

## Methods

### Soil amendments treatments

The field experiment was conducted in west of Zhejiang province in a contaminated red paddy soil. The soil was lightly contaminated by Cd (0.51 mg kg^−1^) and Pb (106.64 mg kg^−1^), which exceeded Chinese Environmental Quality Standards for Soils. Basic properties of the soil are presented in (Table 2) which was analyzed with the standard methods^55,56^. The experiment was started from March/2017and ends at July/2017. Total 11 amendments product were collected from different sources, including lime, DASY, DEK1, Biochar, Fe-Biochar, Yirang, Ca-Mg-P fertilizer, GSA-1, GSA-2, GSA-3, and GSA-4 respectively. Basic physicochemical properties of the amendments are listed in Table 3. The later four amendments were newly made by our group with different organic, inorganic and minerals combination. These amendments products were applied at 1% rate with three replications for each treatment following a randomized complete block design. Plots area of each replicate was 8 m × 8 m with separate inlet and outlet for irrigation and drainage. Overall 12 treatments with one CK were applied before rice seedling transplanting and soil sampling was done before and after different treatment times. Amendments were mixed thoroughly on the upper surface of soil and then ploughed mechanically into 0–20 cm plow layer 15 days before transplanting. Basic nitrogen (N), phosphorus (P as P_2_O_5_), and potassium (K as K_2_O) fertilizers were applied in the form of urea, diammonium phosphate and sulphate of potash, respectively at following rate in all plots (N-P_2_O_5_-K_2_O: 145-60-165 kg ha^−1^). Phosphorus fertilizer was not applied in the treatment where Ca-Mg-P fertilizer was used as amendment/fixing agent.

**Table 2:** Physicochemical properties of the tested soil.

Soil texture	Silty clay
Soil pH	5.69
CEC (Cmolc kg^−1^)	10.40
Organic carbon (g kg^−1^)	71.35
Total Pb (mg kg^−1^)	106.6
Total Cd (mg kg^−1^)	0.51

Sand 10%, Silt 39.70%, Clay 50.30%.

**Table 3:** Physiochemical properties of tested soil amendments tested.

No.	Treatment name	pH	CEC (Cmolc kg^−1^)	Organic carbon (g kg^−1^)	Total Pb (mg kg^−1^)	Total Cd (mg kg^−1^)
1	Lime	11.34	—	N/D	0.692	0.26
2	DASY	11.52	6.92	25.84	3.042	0.24
3	DEK1	9.70	8.17	2.27	3.173	1.03
4	Biochar	8.16	19.21	403	1.696	0.07
5	Fe-Biochar	6.41	20.43	297	1.731	0.03
6	Yirang	11.55	—	7.58	2.343	1.02
7	CaMg-P	7.4	—	N/D	3.150	0.13
8	GSA-1	11.78	148	28.12	6.615	0.14
9	GSA-2	11.7	31.40	31.52	2.505	0.16
10	GSA-3	11.68	—	74.21	4.483	0.24
11	GSA-4	11.72	—	32.31	4.728	0.14

Note: CK (control), Lime, DASY (DaSan Yuan), DEK1 (Di Kang No. 1), Yirang, GSA-1(Green Stabilizing Agent 1), GSA-2 (Green Stabilizing Agent 2), GSA-3(Green Stabilizing Agent 3), and GSA-4 (Green Stabilizing Agent 4).

### Early rice seedling

Early rice cultivar used for experiment was (Zhong zao 39) and seedlings were cultured in early April. All the cultural practices were kept the same for all treatments throughout the experiment. Harvesting was performed at maturity and 15 plants were collected randomly. Plant growth parameters were measured in field at harvesting time. Yield attributes were measured for 1 m^2^ area in each plot. Harvested plant samples were rinsed with deionized water, oven dried and separated into roots, shoots, husk and grains for analysis.

### Soil and plant analyses

Soil samples were collected from zero (transplanting) to three month at the one month interval from rice field. Randomly, five samples were collected from each plot making a composited sample. The collected soil samples were used for determining soil pH and DTPA extractable Cd and Pb. pH was determined by mixing soil with water at 1: 2.5 soil: water ratio by using a pH meter (PB-10, Sartorius, Germany). DTPA extractable heavy metals (Cd and Pb) was determined by mixing 20 g of soil with 50 mL DTPA-TEA solution (0.005 M DTPA, 0.1 M TEA, and 0.01 M CaCl_2_, pH = 7.3). This mixture was then shaken for 2 hrs at 25 °C on a shaker. Suspensions were then passed through 0.45-μm filter membrane and concentration of metals (Cd and Pb) was measured using ICP-MS (Agilent, 7500a, USA)^55,56^.

Dried plant samples were ground for heavy metals analysis^8,57^. Cadmium and Pb in roots, shoots, husk and grains were determined by digestion of 0.200 g plant sample with mixed acid solution (HClO_4_ and HNO_3_) at 170 °C for 4 hrs and cooled at room temperature and diluted with distilled water to make volume to 25 ml. Cadmium and Pb concentration in digested solution was then measured using the ICP-MS^8^.

### Leaf photosynthesis measurement

Photosynthetic rate (Pn) and transpiration rate (Tr) were measured at day 70 of rice transplanting in field using a portable infrared gas analyzer (IRGA).

### Statistical analysis

One way ANOVA was conducted to compare different treatment means using SPSS 20.0 and Origin Pro 8.0. All the results are presented as average of three replicates and standard error was estimated using Microsoft excel 2007.

## Data Availability

The data for this manuscript is available on demand in excel sheet.
